# A cross-sectional study on the prevalence of cardiovascular disease in elderly patients with long-term type 2 diabetes mellitus mainly attended in private clinics in Mexico. The CAPTURE study

**DOI:** 10.1186/s13098-023-01231-6

**Published:** 2023-12-07

**Authors:** José L. Arenas-León, Enrique C. Morales-Villegas, Ernesto G. Cardona-Muñoz, Marco A. Alcocer-Gamba, Juan P. Ramirez-Contreras, Aleida Y. Contreras-Sandoval, Guillermo González-Galvez

**Affiliations:** 1 Hospital Angeles San Luis Potosí, Camino a La Presa 250-850, Col. Burócrata del Estado, CP 78200 San Luis Potosí, S.L.P México; 2 Aguascalientes Cardiometabolic Research Center at MAC Hospital, República de Perú 102-201, C.Las Americas, 20230 Aguascalientes, Ags México; 3 Sierra, Mojada 950, Colonia Independencia, Guadalajara, Jalisco CP 44340 México; 4 Facultad de Medicina, Universidad Autónoma de Querétaro, Clavel 200, Prados de La Capilla, 76176 Santiago de Querétaro, Qro México; 5 Centro de Estudios Clínicos de Querétaro, Instituto de Corazón de Querétaro, Prol. Priv. Ignacio Zaragoza 16B, 2º Piso, Colonia Centro, Querétaro, Qro. Mexico; 6 Novo Nordisk México SA de CV. Homero, 1500, 3Er Floor, Chapultepec, CP 7600011560 Mexico, Mexico; 7 Jalisco Institute of Clinical Research in Diabetes and Obesity, Guillermo Gonzalez-Galvez, MD, FACE, Mexico, Mexico

**Keywords:** Atherosclerotic cardiovascular disease, Cardiovascular disease, Aged, Glucagon-like peptide-1 receptor agonists, Mexico, Non-interventional study, Prevalence, Sodium-glucose co-transporter-2 inhibitors, Type 2 diabetes

## Abstract

**Background:**

To estimate the contemporary prevalence of established cardiovascular disease (CVD) in adults with type 2 diabetes (T2D) in Mexico.

**Methods:**

CAPTURE was a multinational, non-interventional, cross-sectional study across 13 countries from five continents. Standardized demographic and clinical data were collected from adults with T2D attending a single routine healthcare visit in primary or specialized care between December 2018 and September 2019. Data from Mexico are analyzed in this study.

**Results:**

Of the 9,823 patients included in the CAPTURE study, 820 (8.3%) participants were from Mexico, mainly attended in private centers (29.3% in 6 specialized diabetes treatment centers and 70.7% in 26 primary care centers). The median age was 63.0 years, 52.6% were women, the duration of diabetes was 11.8 years and the average HbA1c 7.5%. The weighted prevalence [95% CI] of CVD and atherosclerotic CVD was 36.9% [34.1–39.6] and 29.5% [26.7–32.3], respectively. Additionally, the prevalence of coronary heart disease, heart failure, peripheral arterial disease and cerebrovascular disease was 23.1% [20.6–25-7], 8.4% [6.8–10.0], 5.0% [3.5–6.5] and 3.9% [2.6–5.2], respectively. Glucose lowering drugs were used in 88.5% of patients, being metformin the most commonly drug used (79.4%), followed by sulfonylureas (26.3%). SGLT-2 inhibitors and GLP1 receptor agonists were used in 15.5% and 3.9%, respectively.

**Conclusions:**

In Mexico, nearly four out of ten patients with T2D mainly attended in private centers have CVD, particularly atherosclerotic CVD. Most patients were not taking glucose lowering drugs with proven CV benefit.

## Background.

Type 2 diabetes (T2D) is the main health care problem in Mexico [1]. According to the IDF 2021 Diabetes Atlas, Mexico is in 7th place in the world in the number of people with diabetes, rising from 10.3 million in 2011 to 14.1 million in 2021, and expected to increase to 21.2 million by 2045 [2]. Of note, the prevalence of diabetes in adult population (20–79 years) in Mexico has increased from 15.9% in 2011 to 16.9% in 2021 [2, 3]. On the other hand, it has been calculated that the number of patients with undiagnosed diabetes in 2021 was around 6.7 million (47.5% of all patients with diabetes) [2]. In Mexico, data from the Ensanut 2022 showed that the prevalence of diagnosed and undiagnosed diabetes was 12.6% and 5.8%, respectively, with a total diabetes prevalence of 18.3% [4]. In addition, total diabetes-related health expenditure in 2021 in Mexico was huge, accounting for $19.9 billion [2]. Importantly, the economic burden related to diabetes has risen in the last years, with an estimated increase of 26% [5].

As nearly 20% of the preventable deaths in Mexico are caused by diabetes, and excess mortality associated with previously diagnosed diabetes accounts for one third of all deaths, it is mandatory to improve the prevention, early diagnosis, optimal and holistic management of patients with T2D [1, 6]. In addition- and explained by a late diagnosis, suboptimal and glucocentric management, the death rate in people with diabetes is higher in those patients who also suffer from other conditions, particularly those with renal disease and cardiovascular disease (CVD) [6]. Furthermore, the presence of diabetes doubles the risk of developing CVD [7]. Unfortunately, data regarding the prevalence of CVD in patients with T2D are outdated, not reflecting the current epidemiology of T2D, or have been focused on selected populations or are data come from only Europe or United States [8–10].

Additionally, these studies have not analyzed the impact of novel strategies and therapies. This is important, as in the recent years, different clinical trials have demonstrated that over the standard of care some glucagon-like peptide 1 (GLP-1) receptor agonists and sodium-glucose cotransporter-2 (SGLT2 inhibitors) reduce the risk of CV and renal outcomes in patients with T2D and high CVD risk and/or established CVD and/or chronic kidney disease [11–16]. In this context, recent guidelines recommend that these drugs should be preferentially used in this population [17]. As a result, it is important to ascertain whether these drugs are being used in clinical practice.

CAPTURE (NCT03786406 [Europe] and NCT03811288 [non-Europe]) was a non-interventional, cross-sectional study conducted at 214 centers across 13 countries worldwide (Australasia: Australia; East Asia: China and Japan; Europe: Czech Republic, France, Hungary, and Italy; Latin America: Argentina, Brazil, and Mexico; and the Middle East: Israel, Kingdom of Saudi Arabia and Turkey) performed with the aim to establish the prevalence of the different types of CVD in patients with T2D. In addition, the clinical profile and management of this population, with a particular focus on glucose lowering drugs with proven CV and renal benefit (GLP-1 receptor agonists and SGLT2 inhibitors) were also analyzed [18]. In this study, data from Mexico were determined.

## Methods

The design and methods of the study have been previously published [18]. Briefly, the CAPTURE study consecutively included adult patients, with a diagnosis of T2D at least 6 months before being enrolled that were treated according to routine clinical practice. Patients with type 1 diabetes, known congenital heart disease or malformation, and incapacity to adequately understand the study requirements were excluded from the study. The study in Mexico was approved by the regulatory authorities (Cofepris) and the research and ethics committee of each participating center. All participants provided written informed consent prior to inclusion. A total of 32 mainly private sites (6 specialized diabetes treatment centers and 26 primary care centers) distributed all around Mexico with 91 investigators and assistant investigators participated in the study. Patients were recruited from December 2018 to September 2019.

Data collections were performed during a single, routine visit at each site by the treating physician or a trained delegate using a standardized electronic case report form. Data associated with complications were obtained from interrogation or clinical records. This questionnaire included demographic information, a record of established CVD and the drugs used to treat diabetes and comorbidities. Data were transferred to a central database via a web-based data capture system. Data were collected from both, the clinical history of patients and the interview with the physician.

The clinical profile, glucose-lowering and CV drugs were analyzed in the overall population from Mexico and stratified by CVD, and in the global study data. The overall prevalence estimates of CVD and subtypes were calculated in the Mexico population and in the overall study population. Only current medications or those discontinued within the previous 3 months were recorded. Glucose lowering drugs were further grouped according to demonstrated CV and renal benefit status defined by the 2020 American Diabetes Association guidelines and included three GLP-1 receptor agonists (dulaglutide, liraglutide, and semaglutide) and three SGLT2 inhibitors (canagliflozin, dapagliflozin, and empagliflozin) [19].

Established CVD included patients with cerebrovascular disease, carotid artery disease, coronary heart disease, peripheral artery disease, heart failure, cardiac arrhythmia, or aortic disease. Atherosclerotic CVD included patients with cerebrovascular disease, carotid artery disease, coronary heart disease, or peripheral artery disease, Coronary heart disease was defined as those with myocardial infarction, stable coronary artery disease, other ischemic heart disease, or past revascularization procedure- Heart failure included those patients with symptomatic or asymptomatic heart failure, or hospitalization for heart failure. Cerebrovascular disease was defined as the presence of ischemic, hemorrhagic or unspecified stroke, or transient ischemic attack. Peripheral artery disease was defined as asymptomatic peripheral artery disease (low-ankle branchial index [< 0.90] or pulse abolition), claudication, limb ischemia, or non-traumatic amputation. Cardiac arrhythmia and conduction abnormalities included atrial fibrillation, atrial flutter, supraventricular or ventricular tachycardia, ventricular fibrillation, and bradyarrhythmia (sinus node dysfunction or AV block). Aortic disease was defined as the presence of aortic dissection or aneurysm, or thromboembolic aortic disease.

### Statistical analysis

Results are presented as descriptive statistics: mean (standard deviation), median, minimum, maximum and maximum for continuous variables and percentage for categorical variables (based on information available and taking missing information into account). The prevalence (95% confidence interval [CI]) of established CVD, atherosclerotic CVD, and CVD subtypes were estimated in the population from Mexico and in the overall study population. Overall prevalence estimates were calculated as weighted estimates. Statistical analyses were carried out using SAS, version 9.4 (SAS Institute, Cary, NC, USA).

## Results

### Study sample

Of the 9823 adults with T2D from the overall study sample, 820 (8.3%) participants were included in Mexico. Of these, 240 (29.3%) patients were attended in 6 specialized diabetes treatment centers and 580 (70.7%) in 26 primary care centers.

In Mexico population, 52.6% of patients were women, the median age was 63.0 years, the duration of diabetes 11.8 years and the average HbA1c 7.5%. The average body mass index was 28.8 kg/m^2^, 66.1% of patients had hypertension, and 64% had a glomerular filtration rate less than 90 ml/min/1.73m^2^. With regard to the lipid profile, median total cholesterol was 174 mg/dL, LDL cholesterol 98 mg/dL, HDL cholesterol 42.47 mg/dL and triglycerides166 mg/dL. No differences in the clinical profile were found regarding the clinical setting in which the patients were attended (primary care vs specialized centers): women (52.9% vs 51.7%), age (63.0 vs 62.0 years), median duration of diabetes (11.8 vs 11.0 years), HbA1c (7.6% vs 7.5%) body mass index (28.7 vs 29.3 kg/m^2^), hypertension (67.1% vs 63.6%), glomerular filtration rate less than 60 ml/min/1.73m^2^ (23.2% vs 25.7%). In addition, 32.6% of patients had micro-/macroalbuminuria, 18.5% neuropathy, and 10.1% retinopathy. Regarding the global study data, 45.5% of patients were women, the median age was 64.0 years, the duration of diabetes was 10.7 years and the average HbA1c 7.3%. The average body mass index was 29.0 kg/m^2^, 70.1% of patients had hypertension, and 65.3% had a glomerular filtration rate less than 90 ml/min/1.73m^2^ (Table 1).

**Table 1:** Clinical characteristics of the study population in Mexico classified by status of cardiovascular disease and in the overall study population

	Study population (Mexico) (n = 820; 100%)		By CVD status (Mexico)				Overall study population (n = 9823)	
			CVD (n = 303; 36.9%)		No CVD (n = 517; 63.1%)			
	n	Data	n	Data	n	Data	n	Data
Female, n (%)	820	431 (52.6)	302	135 (44.7)	518	296 (57.1)	9823	4465 (45.5)
Age, years, median [min–max or IQR]	820	63.0 [18–94]	302	66.0 [32–94]	518	61 [18.0–90]	9823	64.0 [56.0–71.0]
Race, n (%)				-				
Caucasian	820	539 (65.7)	302	216 (71.3)	518	323 (62.4)		6487 (66.0)
Asian		1 (0.1)		1 (0.3)		0	9822	2133 (21.7)
Black or African American		0		0		0		158 (1.6)
Other		280 (34.1)		86 (28.4)		194 (37.5)		1044 (10.6)
Diabetes duration, years, median [min–max or IQR]	820	11.77 [0.50–46.76]	302	13.77 [0.54–46.76]	518	10.7 [0.50–43.7]	9811	10.7 [5.6–17.9]
HbA1c, %, median [min–max or IQR]	586	7.5 [4.3–16.3]	219	7.7 [4.3–16.3]	367	7.5 [4.3–16.5]	9104	7.30 [6.60–8.40]
HbA1c, mmol/mol, median [min–max or IQR]	586	58.4 [23.50–154.66]	219	60.66 [23.50–154.66]	367	58.4 [23.5–145.92]	9104	56 [49–68]
FPG, mg/dL-mmol/L, median [min–max or IQR]	662	132 [53–517]	246	126.25 [64–507]	416	134 (53.0–517.0)	8204	7.60 [6.30–9.38]
Body weight, kg, median [min–max or IQR]	820	75.00 [37–145]	302	75.00 [37.0–135.0]	518	75.0 [39.0–145.0]	9742	79.3 [68.7–92.0]
BMI, kg/m2, median [min–max or IQR]	820	28.80 [16.00–50.60]	302	28.5 [16.0–50.60]	518	28.9 [16.3–49.3]	9611	29.0 [25.8–33.1]
Systolic blood pressure, mmHg, median [min–max or IQR]	820	124 [80–200]	302	127.0 [90–198]	518	122.5 [80.0–200.0]	9618	130.0 [120.0–140.0]
Diastolic blood pressure, mmHg, median [min–max or IQR]	820	74 [47–112]	302	73.0 [47–104]	518	74.0 [50.0–112.0]	9616	78.0 [70.0–82.0]
Total cholesterol, mg/dL-mmol/L, median [min–max or IQR]	613	174 [77–492]	234	159.5 [77–492]	379	180.0 [83.0–361.0]	8272	4.34 [3.68–5.14]
LDL cholesterol, mg/dL-mmol/L, median [min–max or IQR]	510	98 [21–245]	191	82.0 [21–210]	319	105.0 [32.20–245.00]	8090	2.39 [1.81–3.08]
HDL cholesterol, mg/dL-mmol/L, median [min–max or IQR]	503	42.47 [17.20–90.00]	195	40.0 [17.3–86.0]	308	44.0 [17.20–90.00]	7965	1.15 [0.98–1.40]
Triglyceride, mg/dL-mmol/L, median [min–max or IQR]	598	166 [48–1860]	229	166.7 [52.4–1860]	369	165.0 [48.0–1400.0]	8466	1.60 [1.13–2.27]
eGFR, mL/min/1.73 m^2^, n (%)		-		-				
> 89	525	189 (36.0)	188	49 (26.1)	337	140 (41.5)	7923	2746 (34.7)
> 59–89		209 (39.8)		82 (43.6)		127 (37.7)		3512 (44.3)
> 29–59		99 (18.9)		48 (25.5)		51 (15.1)		1450 (18.3)
≤ 29		28 (5.3)		9 (4.8)		19 (5.6)		215 (2.7)
Albuminuria^a^, n (%)								
Normal–mildly increased	300	202 (67.3)	103	58 (56.3)	197	144 (73.1)	6482	4338 (66.9)
Microalbuminuria		37 (22.3)		34 (33.0)		33 (16.8)		1607 (24.8)
Macroalbuminuria		31 (10.3)		11 (10.7)		20 (10.2)		537 (8.3)
Medical history of hypertension, n (%)	819	541 (66.1)	302	242 (80.1)	517	299 (57.8)	9643	6756 (70.1)
Familial hypercholesterolemia, n (%)	656	28 (4.3)	230	14 (6.1)	426	14 (3.3)	6634	676 (10.2)
Retinopathy, n (%)		-						
Yes	820	83 (10.1)	302	37 (12.3)	518	46 (8.9)	9818	1455 (14.8)
Yes (mentioned by patient)		41 (5.0)		12 (4.0)		29 (5.6)		399 (4.1)
No		696 (84.9)		253 (83.8)		443 (85.5)		7964 (81.1)
Neuropathy, n (%)		-						
Yes	820	152 (18.5)	820	47 (15.6)	518	66 (12.7)	9817	1774 (18.1)
Yes (mentioned by patient)		54 (6.6)		15 (5.0)		39 (7.5)		459 (4.7)
No		614 (74.9)		240 (79.5)		374 (72.2)		7584 (77.3)
Smoking status,		-						
Current	820	73 (8.9)	302	19 (6.3)	518	54 (10.4)	6178	1322 (13.6)
Previous		247 (30.1)		126 (41.7)		121 (23.4)		2613 (26.9)
Never		500 (61.0)		157 (52.0)		343 (66.2)		5790 (59.5)
Duration of smoking^b^, years, median [min–max or IQR]	320	20.0 [0.0–59.0]	145	20.0 [0.0–57]	175	20.0 [0.00–59.00]	3733	28.0 [15.0–39.0]
Physical activity^c^, days per week, n (%)								
0–1	694	424 (61.1)	256	165 (64.5)	438	259 (59.1)	7492	3599 (48.0)
2–3		91 (13.1)		26 (10.2)		65 (14.8)		1613 (21.5)
4–5		103 (14.8)		27 (10.5)		76 (17.4)		883 (11.8)
6–7		76 (11.0)		38 (14.8)		38 (8.7)		1397 (18.6)

Qualitative variables are defined as n (%); Quantitative variables are defined as median [minimum; maximum] (Mexico) or [IQR] (overall population. To convert glucose values to mg/dL, divide by 0.0555. To convert cholesterol values to mg/dL, divide by 0.0259. To convert triglyceride values to mg/dL, divide by 0.0113

^a^Defined as: normal–mildly increased, urinary excretion < 30 mg/24 h or UACR < 30 mg/g; microalbuminuria, urinary excretion 30–299 mg/24 h or UACR 30–299 mg/g; macroalbuminuria, urinary excretion ≥ 300 mg/24 h or UACR ≥ 300 mg/g

^b^ Only applies to participants categorized as current or previous smokers

^c^ Days with ≥ 30 min of moderate activity

*BMI* body mass index, *CVD* cardiovascular disease, *eGFR* estimated glomerular filtration rate, FPG fasting plasma glucose, *HbA1c* glycated hemoglobin, *HDL* high density lipoprotein, *IQR* interquartile range, *LDL* low-density lipoprotein, *UACR* urinary albumin to creatinine ratio

Additionally, the clinical characteristics of the Mexico population were stratified according to CVD status. Compared to the non-CVD subgroup, the CVD subgroup was numerically older (66.0 vs 61.0 years), less commonly women (44.7% vs 57.1%), had a higher diabetes duration (13.8 vs 10.7 years), higher HbA1c levels (7.7% vs 7.5%), more hypertension (80.1% vs 57.8%), kidney dysfunction (estimated glomerular filtration rate < 60 mL/min/1.73 m^2^: 30.3% vs 20.7%), micro-/macroalbuminuria (43.7% vs 27.0%), neuropathy (15.6% vs 12.7%) and retinopathy (12.3% vs 8.9%) (Table 1).

### CVD prevalence

**Fig. 1:**
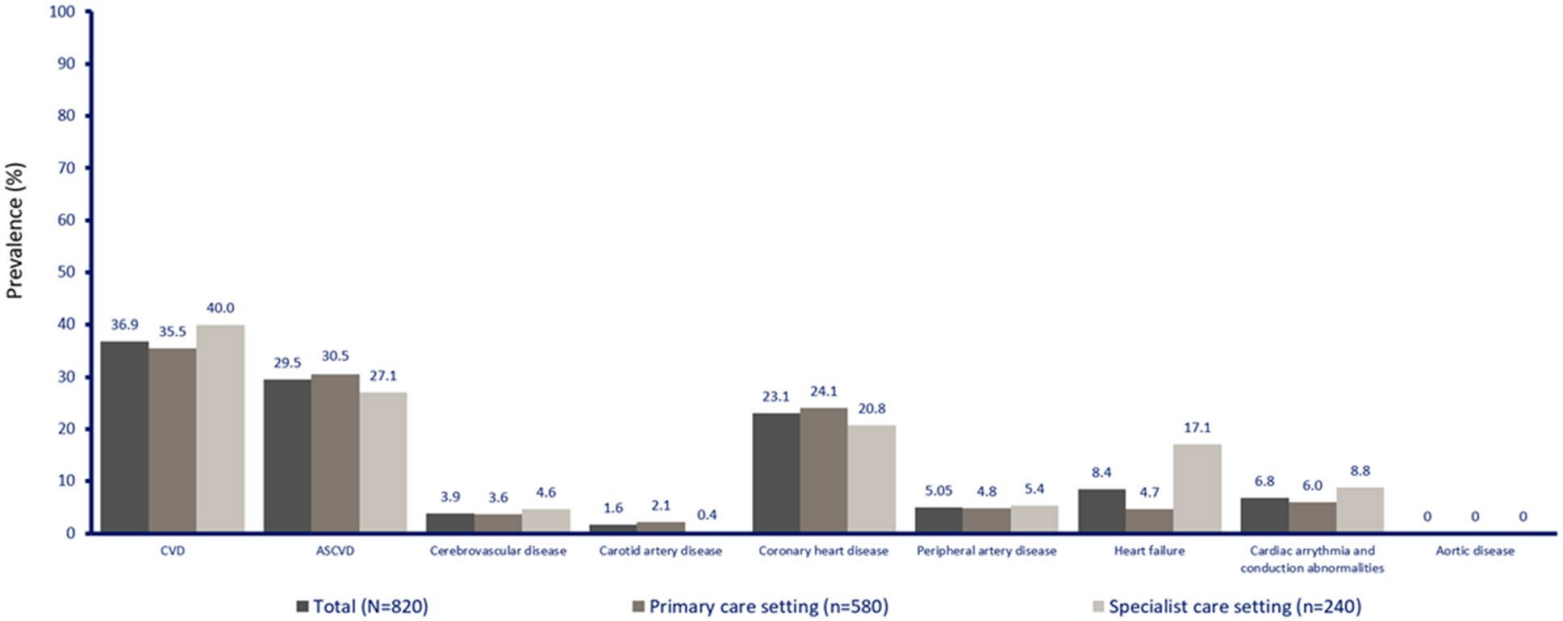
Prevalence of CVD subtype stratified by care setting in Mexico. ASCVD: atherosclerotic cardiovascular disease; CVD: cardiovascular disease

In the CAPTURE Mexico population, the weighted prevalence [95% CI] of CVD was 36.9% [34.1–39.6] and the weighted prevalence of atherosclerotic CVD was 29.5% [26.7–32.3]. Additionally, the prevalence of coronary heart disease, heart failure, peripheral arterial disease and cerebrovascular disease was 23.1% [20.6–25-7], 8.4% [6.8–10.0], 5.0% [3.5–6.5] and 3.9% [2.6–5.2], respectively (Table 2). The prevalence of these conditions differed according to the clinical setting in which the patients were attended (primary care vs specialized centers CV disease (35.5% vs 40.0%), atherosclerotic CVD (30.5% vs 27.1%), coronary heart disease (24.1% vs 20.8%), heart failure (4.7% vs 17.1%), peripheral arterial disease (4.8% vs 5.4%) and cerebrovascular disease (3.6% vs 4.6%) (Fig. 1). Regarding the global study data, the weighted prevalence of CVD was 34.8% [32.7–36.8] and the weighted prevalence of atherosclerotic CVD was 31.8% [29.7–33.8]. Additionally, the prevalence of coronary heart disease, heart failure, peripheral arterial disease and cerebrovascular disease was 17.7% [16.2–19.3], 2.4% [2.1–2.7], 2.6% [2.0–3.1] and 7.2% [5.9–8.4], respectively (Table 2).

**Table 2:** Overall prevalence estimates of CVD in adults with T2D in the overall study population (n = 9823) and in Mexico (n = 820)

	Mexico		Overall study population	
CVD diagnosis	Number of patients	Prevalence, % [95% CI]	Number of patients	Prevalence†, % [95% CI]
CVD	302	36.9 [34.1–39.6]	3582	34.8 [32.7–36.8]
Atherosclerotic CVD	242	29.5 [26.7–32.3]	3074	31.8 [29.7–33.8]
Arterial coronary disease	189	23.1 [20.6–25-7]	2078	17.7 [16.2–19.3]
Arterial carotid disease	13	1.6 [0.9–2.3]	627	8.4 [7.0–9.7]
Cerebrovascular disease	32	3.9 [2.6–5.2]	672	7.2 [5.9–8.4]
Cardiac arrhythmia and conduction disorders	56	6.8 [5.2–8.5]	685	4.2 [3.4–5.1]
Arterial peripheral disease	16	5.0 [3.5–6.5]	489	2.6 [2.0–3.1]
Heart failure	69	8.4 [6.8–10.0]	579	2.4 [2.1–2.7]
Aortic disease	0	0	96	0.4 [0.2–0.6]

^†^Weighted estimate across Argentina, Australia, Brazil, China, Czech Republic, France, Hungary, Israel, Italy, Japan, Mexico, Saudi Arabia and Turkey

CVD: cerebrovascular disease, carotid artery disease, CHD, peripheral artery disease, heart failure, cardiac arrhythmia, aortic disease; Atherosclerotic CVD: cerebrovascular disease, carotid artery disease, CHD, peripheral artery disease; CHD: myocardial infarction, stable coronary artery disease, other ischemic heart disease, past revascularization procedure; Heart failure: symptomatic or asymptomatic heart failure, hospitalization for heart failure; Cerebrovascular disease: ischemic, hemorrhagic or unspecified stroke, transient ischemic attack; Peripheral artery disease: asymptomatic peripheral artery disease (low-ankle branchial index [< 0.90] or pulse abolition), claudication, limb ischemia, non-traumatic amputation; Cardiac arrhythmia and conduction abnormalities: atrial fibrillation, atrial flutter, supraventricular or ventricular tachycardia, ventricular fibrillation, bradyarrhythmia (sinus node dysfunction or AV block); Carotid artery disease; Aortic disease: aortic dissection or aneurysm, thromboembolic aortic disease

*AV* atrioventricular, *CHD* coronary heart disease, *CI* confidence interval, *CVD* cardiovascular disease, *T2D* type 2 diabetes

### Use of glucose-lowering drugs

**Fig. 2:**
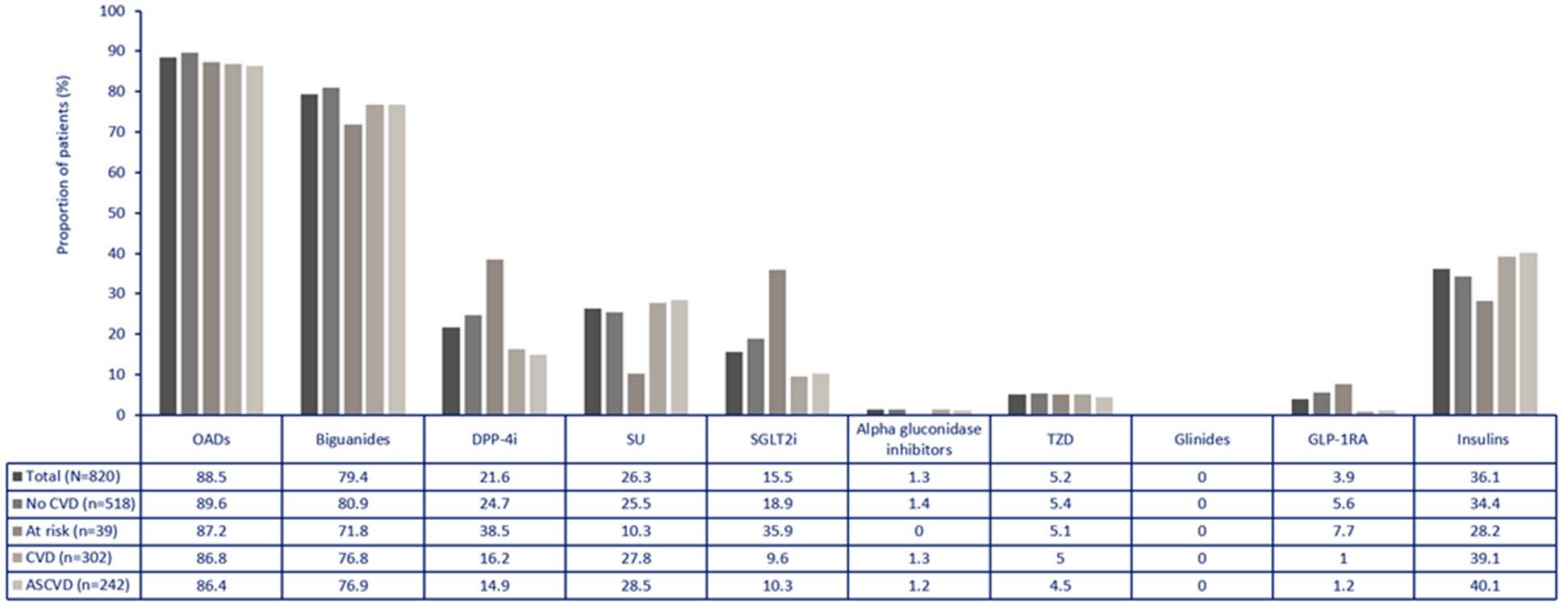
Use of glucose lowering drugs stratified by medication class and CVD subtype in Mexico. ASCVD: atherosclerotic cardiovascular disease; CVD: cardiovascular disease; DPP-4: dipeptidyl peptidase 4; GLP-1RA: glucagon-like peptide-1 receptor agonist; OAD: oral anti-diabetic drug; SGLT2i: sodium-glucose co-transporter-2 inhibitor; SU: sulphonylureas; TZD: thiazolidinediones. Population at risk was defined according to REWIND and DECLARE-TIMI 58 criteria [10, 14]

**Fig. 3:**
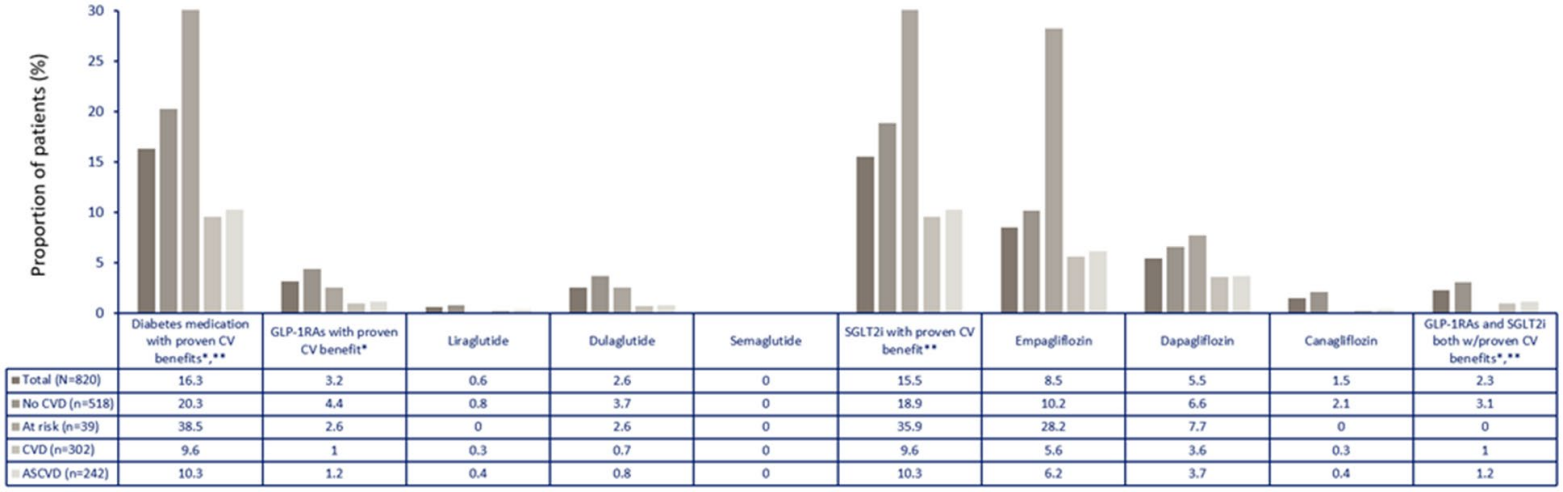
Use of glucose lowering drugs with proven CV benefit stratified by CVD subtype in Mexico. *Liraglutide, semaglutide, dulaglutide **Empagliflozin, canagliflozin, dapagliflozin. ASCVD: atherosclerotic cardiovascular disease; CV: cardiovascular; CVD: cardiovascular disease; GLP-1RA: glucagon-like peptide-1 receptor agonist; SGLT2i: sodium-glucose co-transporter-2 inhibitor. Population at risk was defined according to REWIND and DECLARE-TIMI 58 criteria [10, 14]

In the total CAPTURE Mexico sample, glucose lowering drugs were used in 88.5% of patients, without differences according to CVD status (86.8% vs 89.6%) and slightly lower than in the overall study population (96.6%). Metformin was the most common oral glucose-lowering drug used (79.4%) in Mexico, followed by sulfonylureas (26.3%), DPP-4 inhibitors (21.6%) and SGLT-2 inhibitors (15.5%). GLP1 receptor agonists were used in 3.9% of patients. Insulin was prescribed in 36.1% of patients and monotherapy in 32.3% of participants. Only 16.3% of patients received any glucose lowering drug with proven CV benefit (vs 21.9% in the global study sample), more commonly in patients without CVD (9.6% vs 20.3%) (Table 3 and Figs. 2 and 3).

**Table 3:** Use of glucose-lowering drugs in Mexico categorized by cardiovascular disease and in the overall study population

	Study population N = 820	CVD status		Overall study population (n = 9823)
		CVD n = 302	No CVD n = 518	
Any glucose lowering drug	88.5%	86.8%	89.6%	96.6%
Any glucose lowering drug with proven CV benefit*	16.3%	9.6%	20.3%	21.9%
Oral glucose lowering drugs				
Biguanide	79.4%	76.8%	80.9%	75.6%
DPP-4 inhibitor	21.6%	16.2%	24.7%	29.2%
Sulfonylurea	26.3%	27.8%	25.5%	21.6%
SGLT-2 inhibitor	15.5%	9.6%	18.9%	16.0%
Alpha glucosidase inhibitor	1.3%	1.3%	1.4%	5.3%
Thiazolidinedione	5.2%	5%	5.4%	3.8%
Glinide	0	0	0	3.3%
Others	0	0	0	0.3%
Insulin	36.1%	39.1%	34.4%	37.7%
GLP-1 receptor agonist (arGLP-1)	3.9%	1.0%	5.6%	10.1%
Monotherapy	32.3%	36.8%	29.7%	30.0%
Dual therapy†	46.5%	45.7%	46.9%	36.3%
Triple therapy†	15.9%	14.2%	16.8%	22.3%
Treatment with ≥ 4 glucose lowering drugs	4%	1.3%	5.6%	8.0%

insulin, biguanide and sulfonylurea and insulin, biguanide and DPP-4 inhibitor and SGLT-2 inhibitor. †Reported in ≥ 1.0% of the total population of the CAPTURE study, group with CVD or group without CVD; * defined per 2020 American Diabetes Association guidelines as GLP-1 RAs: dulaglutide, liraglutide, and semaglutide; and SGLT2is: canagliflozin, dapagliflozin, and empagliflozin

*CVD* cardiovascular disease, *iDPP-4* dipeptidyl peptidase 4 inhibitor, *arGLP-1 RA* receptor agonist of the peptide similar to type 1 glucagon, *iSGLT-2* type 2 sodium-glucose co-transporter inhibitor

Monotherapy included any of the following drugs: biguanide, insulin, DPP-4 inhibitor, sulfonylurea, SGLT-2 inhibitor or GLP-1 receptor agonist; Dual therapy included any of the following combinations: biguanide and insulin (either), biguanide and DPP-4 inhibitor, biguanide and sulfonylurea, biguanide and SGLT-2 inhibitor, biguanide and arGLP-1, SGLT-2 inhibitor and insulin; Triple therapy included any of the following combinations: biguanide and sulfonylurea and DPP-4 inhibitor, biguanide and DPP-4 inhibitor and insulin (any), biguanide and arGLP-1 and insulin, biguanide and SGLT-2 inhibitor and

### Use of CV drugs

Antihypertensive drugs and other CVD agents were used by 63.3% of the overall Mexico sample, more frequently in those patients with CVD (84.8% vs 50.8%) and similarly than in the overall study population (61.4%). Lipid lowering drugs and platelet aggregation inhibitors were used in 48.2%, particularly statins, and 30.1% of the Mexico population, respectively, more commonly prescribed among patients with CVD (49.6% vs 47.6% and 63.2% and 10.8%, respectively). These numbers were 54.1% and 33.5%, respectively for the global study sample (Table 4).

**Table 4:** Use of cardiovascular drugs in Mexico categorized by cardiovascular disease and in the overall study population

	Study population N = 820	CVD status		Overall study population (n = 9823)
		CVD n = 302	No CVD n = 518	
Antihypertensive drugs and other CVD agents	63.3%	84.8%	50.8%	61.4%
Lipid lowering drugs	48.2%	49.6%	47.6%	54.1%
Statins	43.4%	44.2%	43.1%	51.0%
Ezetimibe	4.8%	7.1%	3.8%	NA
PCS9 inhibitors	0.1%	0	0.2%	NA
Platelet aggregation inhibitors	30.1%	63.2%	10.8%	33.5%
Anticoagulants	3.4%	7.9%	0.8%	4.3%
Diuretics	24.8%	37.4%	17.4%	22.4%

Antihypertensive drugs and other CVD agents included any of the following drugs: angiotensin II blocker, angiotensin converter enzyme inhibitor, calcium channel blocker, beta blocker, combination of alpha and beta blocker, neprilysin inhibitor, or others; lipid lowering drugs included any of the following drugs: statin, ezetimibe, fibrate, PCSK9 inhibitor, nicotinic acid and derivatives, or others; platelet aggregation inhibitors included any of the following drugs: acetylsalicylic acid, P2Y12 inhibitors or dual anti-platelet therapy; anticoagulants included any of the following drugs: vitamin K antagonists, direct oral anticoagulants, heparin; diuretics included any of the following drugs: thiazide, thiazide like diuretic, loop diuretic, aldosterone antagonist, or others. *CVD* cardiovascular disease, *PCSK9* proprotein convertase subtilisin/kexin type 9

## Discussion

Our study showed in a wide sample of patients with long duration T2D attended in primary care or specialist care settings in Mexico that the overall weighted prevalence of CVD was estimated at 36.9% (vs 34.8% in the overall study population), being atherosclerotic CVD the main determinant (79.9% in Mexico vs 85.8% in the global study data). Despite that, only 16.3% of patients with T2D in Mexico were taking glucose lowering drugs with proven CV benefit (vs 21.9% in the global study). Many reasons could explain the underuse of these drugs, and may include access barriers, lack of prescription, underestimation of CV risk, insufficient training, or therapeutic inertia, among others [8, 20–22].

CAPTURE represents the first multinational and standardized study to accurately estimate CVD prevalence in adults with T2D, with a total of 13 participating countries from 5 continents, providing relevant information of different countries around the world, beyond US population. In addition, the generalizability of the findings was high, as the CAPTURE study had few inclusion and exclusion criteria, patients were enrolled consecutively at routine clinic visit and final large global T2D populations were enrolled, allowing CV subgroup analyses [18]. Therefore, the information provided from this study may facilitate the development of specific strategies to reduce CV burden in patients with T2D in Mexico, with the potential implication of physicians and patients, but also local healthcare payers and policy makers.

Mexico recruited 8.3% (n = 820/9823) of the total population of the CAPTURE study, 29% from specialized diabetes treatment centers and 71% from primary care setting. Median age was 63 years, 53% of patients were women and patients had many comorbidities and microvascular complications, without significant differences according to the clinical setting in which patients were attended. This is in line with those studies that have reported that the great majority of patients with long term duration T2D have a high or very high CV risk [23]. In addition, although the differences in the clinical profile according to CVD status were not formally tested, patients with CVD were older, more commonly men and had more CV risk factors and microvascular complications (eg, kidney dysfunction, micro-/macroalbuminuria, neuropathy and retinopathy). In the multinational sample, despite the duration of diabetes was lower, the prevalence of hypertension, current smokers, renal dysfunction, micro-macroalbuminuria, and retinopathy were higher. Therefore, as these differences can have a relevant impact on the management and outcomes of patients with T2D, it is important to perform specific analysis by country.

In the case of Mexico, patients were recruited from specialized diabetes treatment centers (29%) and primary care centers (71%), fundamentally belonging to the private sector, but not the public system. Data from Ensanut 2022 showed that among those patients attended by the health care system, 44% received care in public assistance services [24], but this number decreased to less than 20% in the case of chronic diseases, such as T2D [25, 26]. Of note, private health insurance covers nearly 8% of the population, many of whom are higher-income individuals. Private practice is highly fragmented, and many private services are paid out-of-pocket fee-for-service [25, 26]. Additionally, the Mexican population typically inherits a mix of European, Native American, and African ancestry and this corresponds to the population included in our study (two thirds were Caucasians and one third belonged to other races). As a result, considering all these points, our data may be representative of those patients mainly attended in the private stetting in Mexico, specifically those aged > 60 years with a time of evolution of T2D > 10 years, providing interesting data and a relevant picture about the clinical profile and how these patients are attended in daily clinical practice in Mexico.

Our study showed that median HbA1c was 7.5%, compared to 7.3% in the international global sample. Previous studies have shown that in Mexico around 40% of patients achieve adequate glycemic control [27–29]. The early glycemic control is warranted to reduce the risk of developing micro- and probably macrovascular events in patients with T2D, particularly with the use of those drugs that are not associated with hypoglycemia, in order to avoid the risk of complications [30, 31]. Therefore, more efforts should be performed to reduce the gap between the recommended HbA1c targets by guidelines and clinical practice [32].

In the CAPTURE Mexico population, the weighted prevalence of CVD and atherosclerotic CVD was 36.9% and 29.5%, respectively (vs 34.8% and 31.8% in the multinational sample). Although the numbers found in the overall CAPTURE population were in line with those reported in a systematic literature review of 57 studies until 2015, from 25 countries involving over 4.5 million adults with T2D [10], the figures found in Mexico were quite higher, probably reflecting the mentioned late diagnosis and suboptimal and glycocentric (no holistic) management in addition with very low use of glucose lowering drugs with CV and renal protection. This is important, as diabetes-related deaths are growing, even after the onset of the COVID-19 pandemic [33–35] and the early prescription of CV protective drugs should be a priority in this population [17, 31, 36, 37].

The prevalence of heart failure in this study reached 8% of T2D population. Heart failure is common in patients with T2D, is associated with great morbidity and mortality and can occur in the early stages of the evolution of the disease [38]. As a result, the prompt diagnosis and optimal treatment is mandatory to reduce the risk of complications in this population through the early implementation of those drugs that have demonstrated to reduce HF burden, including SGLT-2 inhibitors [39]. In addition, clinical trials have demonstrated that SGLT-2 inhibitors reduce the risk of developing heart failure in patients with T2D [14–16].

In the overall CAPTURE Mexico sample, glucose lowering drugs were used in 88.5% of patients (vs 97% in the overall study population). Metformin was the most common oral glucose-lowering drug used (79%), followed by sulfonylureas (26%), DPP-4 inhibitors (22%) and SGLT-2 inhibitors (15.5%). Only 16.3% of patients received any glucose lowering drug with proven CV benefit (vs 22% in the global study sample). Patients with T2D should be treated with the double target of achieving HbA1c goals and the use of glucose lowering drug with proven CV and renal benefit, particularly GLP-1 receptor agonists and SGLT-2 inhibitors, with or without metformin [17, 37, 40] -in fact, a recent study performed in Mexico has shown that the inadequate control of diabetes is associated with an increased risk of premature death and CV complications [41]-, unfortunately, as our study showed, this percentage was markedly low in Mexico, even worse than in the general population of the CAPTURE study. To reduce the morbidity and death rate of patients with T2D, the use of these drugs should be enhanced, likely promoted not only by doctors, but also by health service providers and governments. One of the reasons of the low use of GLP-1 receptor agonists despite their proven CV benefit is due to their injectable route of administration [17, 42]. In this context, the introduction of oral semaglutide could be of help [43, 44]. Moreover, strengthening the need of prescribing these drugs in clinical practice through ongoing medical education and empowerment of patients seems mandatory. In addition, considering that glucose lowering drugs with CV and renal protection are now partially or totally reimbursed, it is expected a higher prescription of these drugs, thus translating into a reduction of CV and renal events. Future studies are mandatory to confirm this point.

Treatment of T2D should not be reduced to metabolic control, but to the comprehensive and optimal management and control of all CV risk factors. Thus, a multifactorial intervention on different CV risk factors among patients with T2D has been associated with a marked reduction of mortality, even after several years of follow-up [45, 46]. In the Mexico population of the CAPTURE study, only in two thirds of patients were taking antihypertensive drugs and other CVD agents and nearly half of patient lipid lowering drugs. Remarkably, in patients with CVD, only 63% of patients were taking antiplatelets and 66% lipid lowering drugs, despite median LDL cholesterol was 82 mg/dL (target < 55 mg/dL in patients with atherosclerotic CVD and < 70 mg/dL in high risk patients without atherosclerotic CVD) [36]. Once again, there is an important treatment gap with the use of evidence-based therapies that should be improved.

The study has certain limitations. Since data associated with complications were obtained from interrogation or clinical records, some complications could be underrepresented. On the other hand, prevalence data may have been overestimated as people with complications have the habit of seeing their healthcare services provider more often than the general population with T2D. This may explain why prevalence between primary sites and specialized sites is very similar. Use of drugs may have been influenced by prescribing trends at each site and the possibility that patients depend on the drugs that health institutions provide as a function of a basic list of drugs and that some drugs are only prescribed by specialist doctors. There may also be bias when selecting sites that were selected by a contract research organization employed by the sponsor and approved by staff of the sponsor to ensure that data on CVD is gathered accurately. In addition, CAPTURE collection data was contemporary to the EASD/ADA 2018 guidelines publication [31]. Consequently, the short time between the publication of these recommendations and clinical application was short and by sure this had an impact on the results. As a result, it would be interesting to review these data in the next future to analyze the long-term impact.

## Conclusions

In conclusion, in Mexico nearly four out of ten elderly patients with long duration T2D have CVD, particularly atherosclerotic CVD. Most patients were not taking glucose lowering and CV drugs with proven CV benefit, even less than in the overall study population, leading to a higher risk of complications. Therefore, the CAPTURE study shows that in Mexico there is room for improvement to reduce the gap between guidelines and clinical practice. We propose a medical ecosystem funded on evidence, personalization, empowerment and facilitated access. This ecosystem which includes doctors, patients, families and society -big-pharma, government and other stakeholders- has proven success and improvement of the prognosis of people living with T2DM [47–49].

## Data Availability

The data that support the findings of this study are available from the corresponding author upon reasonable request.
